# Outcomes of hip reconstruction in ambulatory patients with cerebral palsy and spastic hip displacement: a retrospective study of 73 hips in 55 consecutive patients

**DOI:** 10.2340/17453674.2026.45513

**Published:** 2026-02-23

**Authors:** Kyeong Hyeon PARK, Byoung Kyu PARK, Isaac RHEE, Kun Bo PARK, Hoon PARK, Yun Ho ROH, Hyun Woo KIM

**Affiliations:** 1 Division of Pediatric Orthopedic Surgery, Severance Children’s Hospital, Yonsei University College of Medicine, Seoul, Republic of Korea; 2 Department of Orthopedic Surgery, Haeundae Paik Hospital, Inje University College of Medicine, Busan, Republic of Korea; 3 Department of Orthopaedics, St Vincent’s Hospital, Melbourne, Australia; 4 Department of Orthopedic Surgery, Gangnam Severance Hospital, Yonsei University College of Medicine, Seoul; 5 Biostatistics Collaboration Unit, Department of Biomedical Systems Informatics, Yonsei University College of Medicine, Seoul, Republic of Korea

## Abstract

**Background and purpose:**

Currently, there is no specific surgical treatment strategy established for spastic hip displacement in ambulatory patients with cerebral palsy. We aimed to evaluate the outcomes of our hip reconstructions, specifically designed to address hip displacement within the context of single-event multilevel surgery (SEMLS).

**Methods:**

We conducted a retrospective study on patients with Gross Motor Function Classification System (GMFCS) levels II (n = 27) and III (n = 28). Surgical procedures involved various combinations of open or closed reduction, iliac osteotomy, proximal femoral derotational osteotomy, distal femoral derotational and shortening osteotomy, and proximal femoral varus derotational osteotomy. The overall developmental status of the hip was assessed using the Melbourne Cerebral Palsy Hip Classification Scale (MCPHCS) at a mean age of 17.3 years (SD 4.7).

**Results:**

73 hips in 55 patients were included. They underwent hip reconstruction at a mean age of 9.7 years (SD 2.6). 69 of 73 hips achieved successful outcomes. Before surgery, 51 hips had a migration percentage (MP) of 30–60%, 21 had 60–100%, and 1 hip > 100%. At the final follow-up, 12 hips were classified as MCPHCS grade 1 (MP < 10%), 36 as grade 2 (10–15%), 21 as grade 3 (15–30%), 3 as grade 4 (30–60%), and 1 as grade 5 (60–100%). 52 patients either maintained or improved their preoperative GMFCS levels.

**Conclusion:**

Within the SEMLS framework, our tailored hip reconstruction achieved satisfactory hip outcomes in 69 of 73 hips and resulted in sustained improvement in hip stability at long-term follow-up.

The risk of hip displacement in patients with cerebral palsy (CP) is directly related to their gross motor function as graded with the Gross Motor Function Classification System (GMFCS) [1]. Although spastic hip diseases are less frequent in ambulatory patients, the incidence of hip displacement is still notable, with reported rates of 15.1% in GMFCS level II and 41.3% in level III [2]. In terms of motor type and topographical distribution, spastic diplegics have an incidence of 19.2% [2], and late progression of hip displacement can still occur in hemiplegics [3,4]. Recently, Miller et al. suggested that patients with “asymmetric” diplegia classified as levels II and III may experience hip displacement rates different from those typically expected for their respective GMFCS levels [5].

One-stage hip reconstruction, including adequate soft tissue release and femoral varus derotational osteotomy, with or without iliac osteotomy, is a widely accepted treatment for spastic hip displacement. However, previous studies have primarily focused on nonambulatory patients, with little attention to ambulatory individuals [6-8]. The studies only included a small number of ambulatory patients within heterogeneous cohorts. Furthermore, reconstruction techniques typically designed for nonambulatory patients were applied, despite differences in GMFCS levels and degrees of hip displacement.

Gross motor function often deteriorates with age [1], and hip displacement in ambulatory patients likely exacerbates this decline [3,5,9,10]. Consequently, surgical treatment for patients at GMFCS levels I–III should not only focus on reducing the hip but also aim to preserve or enhance their walking abilities [11]. Traditional hip reconstruction used for nonambulatory individuals may not be universally applicable to ambulatory patients, as they must account for altered hip joint biomechanics and concomitant musculoskeletal issues in other joints within the context of walking. We investigated the long-term outcome of our personalized hip approach, implemented within single-event multilevel surgery (SEMLS), in achieving and sustaining improvements in hip condition through a successful hip reconstruction.

## Methods

### Study design and patients

Our hospital’s institutional review board approved this retrospective study and waived the requirement for informed patient consent. The study was conducted and reported in accordance with the STROBE guidelines for cohort studies.

### Surgical techniques

Our surgical techniques were individualized for each patient, taking into account any concomitant knee issues. All patients showed varying degrees of spasticity in their hip flexors, adductors, and knee flexors, along with increased range of hip internal rotation. Preoperatively and at each stage of surgical intervention, we evaluated the degree of soft tissue contractures around the hip and knee joints, alterations in the proximal femoral shape, and concentricity of the femoral head in relation to acetabular deficiency. Ultimately, the specific surgical technique for each hip was determined based on the intraoperative findings.

**Figure 1:**
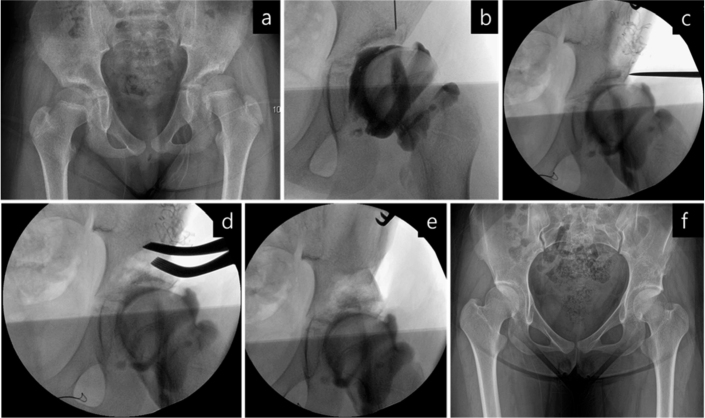
An illustrative case of spastic hip subluxation in a 9-year-old girl. (a) The preoperative anteroposterior pelvic radiograph shows left hip subluxation. (b) The arthrogram confirms dye pooling in the medial joint space. (c) The femoral head was reduced with the hip internally rotated 20° and abducted 15°. (d, e) An iliac osteotomy was performed and a concentric reduction of the hip was achieved without undergoing a simultaneous femoral osteotomy. (f) Final radiograph shows a well-developed hip joint.

We began by releasing all soft tissue contractures around the hip, occasionally extending the release to the distal hamstrings. In 8 select cases (Figure 1), adequate hip reduction was achieved through soft tissue release combined with femoral and/or acetabular osteotomies, as confirmed by intraoperative dynamic arthrography. Severely subluxated hips were managed using traditional open reduction via an anterior approach. In contrast, most hips with an MP < 60% underwent limited anterolateral capsulotomy to assess the degree of reduction at each stage of the trial for anatomic repositioning of the femoral head. We directly visualized the femoral head within the joint and used intraoperative fluoroscopy to supplement the assessment of its position. This combined approach enabled precise adjustments of the hip in the transverse, coronal, and sagittal planes to achieve concentric reduction and guided the selection of the most appropriate surgical procedure for each patient. The capsule was typically closed with non-absorbable sutures. Postoperatively, all patients were immobilized in a hip spica cast for 6 weeks, after which they participated in a comprehensive in-hospital rehabilitation program. Details of concomitant procedures performed at the knees, ankles, and feet, along with subsequent follow-up operations needed for growth-related pathologies, are summarized in Tables 1 and 2.

**Table 1:** Concomitant procedures performed at the time of hip reconstruction

Procedures	Number of limbs	
	ipsilateral	contralateral
Adductors release	–	8
Psoas tendon recession	–	1
Proximal rectus femoris release	2	1
Proximal femoral derotational osteotomy	–	1
Hamstring lengthening	31	10
Rectus femoris transfer	1	1
Anterior distal femoral epiphysiodesis	–	1
Patellar tendon advancement	16	10
Tibial tuberosity epiphysiodesis	9	5
Distal tibial derotational osteotomy	7	2
Gastrocnemius–soleus lengthening	53	25
Planovalgus foot deformity correction	32	16
Equinovarus foot deformity correction	2	1

**Table 2:** Subsequent procedures performed during follow-up

Procedures	Number of limbs	
	ipsilateral	contralateral
Adductors release	10 (6 **^a^**)	2
Psoas tendon recession (ipsilateral) Iliac osteotomy	2	–
Proximal femoral derotational osteotomy	1 (1 **^a^**)	
Proximal femoral varus derotational osteotomy	–	1
Hamstring lengthening	11 (1 **^a^**)	3
Distal femoral extension osteotomy	19 (13 **^a^**)	4 (4 **^a^**)
Anterior distal femoral epiphysiodesis	3	1
Patellar tendon advancement	12	3
Tibial tuberosity epiphysiodesis	9 (2 **^a^**)	2
Proximal tibial medial epiphysiodesis	1	–
Distal tibial derotational osteotomy	2	
Gastrocnemius–soleus lengthening	6 (2 **^a^**)	4 (2 **^a^**)
Tibialis anterior transfer	1	
Tibialis posterior lengthening	2	1
Planovalgus foot deformity correction	4 (1 **^a^**)	1
Epiphysiodesis for limb length discrepancy	1	–

a Number of revision procedures for growth-related recurrence of the corresponding deformity.

Based on the operative strategies employed, we classified the displaced hips in our cohort into 4 groups (see Figure 6). One patient underwent bilateral hip reconstruction, necessitating different surgical techniques for each side. Consequently, this patient was included in 2 different groups (Group A and Group B), resulting in a total of 56 group assignments among 55 patients.

#### Group A: Closed (3 hips) or open reduction (10 hips) with iliac osteotomy (13 hips in 10 patients)

**Figure 2:**
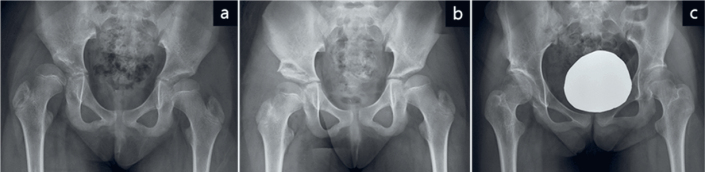
Anteroposterior pelvic radiographs of a patient in Group A. (a) Preoperative radiograph at 10 years and 2 months of age showing right hip subluxation with a migration percentage of 72%. (b) Postoperative radiograph demonstrating reduction of the femoral head and correction of acetabular dysplasia following open reduction and iliac osteotomy. Simultaneous bilateral procedures, including medial hamstring lengthenings, gastrocnemius–soleus lengthenings, and planovalgus foot deformity corrections, were performed. (c) At 14 years and 5 months of age, the Melbourne Cerebral Palsy Hip Classification Scale was graded as 3, and the preoperative Gross Motor Function Classification System level II was maintained.

This reconstruction was performed when the femoral head was concentrically reduced with the hip mildly internally rotated and abducted (Figure 2). A Dega-type iliac osteotomy was performed to ensure adequate acetabular coverage. In 1 patient, guided growth surgery using a transphyseal screw was performed bilaterally to correct coxa valga. The medial hamstrings were lengthened in 8 patients.

#### Group B: Open reduction and proximal femoral derotational osteotomy (FDO) with or without iliac osteotomy (11 hips in 10 patients)

**Figure 3:**
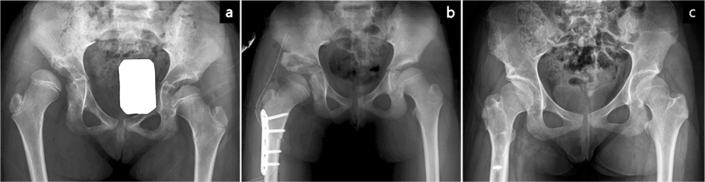
Anteroposterior pelvic radiographs of a patient in Group B. (a) The preoperative radiograph, taken at 9 years and 1 month of age, demonstrates right hip subluxation with a migration percentage of 92%. (b) The postoperative radiograph shows successful reduction of the femoral head and correction of acetabular dysplasia following open reduction, proximal femoral derotational osteotomy, and iliac osteotomy. Additionally, simultaneous bilateral procedures were performed, including medial hamstring lengthening, gastrocnemius–soleus lengthening, and correction of planovalgus foot deformity. (c) At 14 years and 2 months of age, the Melbourne Cerebral Palsy Hip Classification Scale was graded as 3. The patient’s preoperative Gross Motor Function Classification System level III improved to level II.

When intraoperative examination showed that a significant amount of hip internal rotation (> 30°) and no or minimal abduction was needed to achieve concentric reduction of the femoral head, a FDO was performed (Figure 3). This included either an intertrochanteric or subtrochanteric FDO without additional shortening, with an iliac osteotomy being performed concurrently in all but 1 hip. Hamstring lengthenings were done in 7 patients.

#### Group C: Closed (5 hips) or open reduction (24 hips) and distal femoral derotational and shortening osteotomy (FDSO) with iliac osteotomy (29 hips in 22 patients)

**Figure 4:**
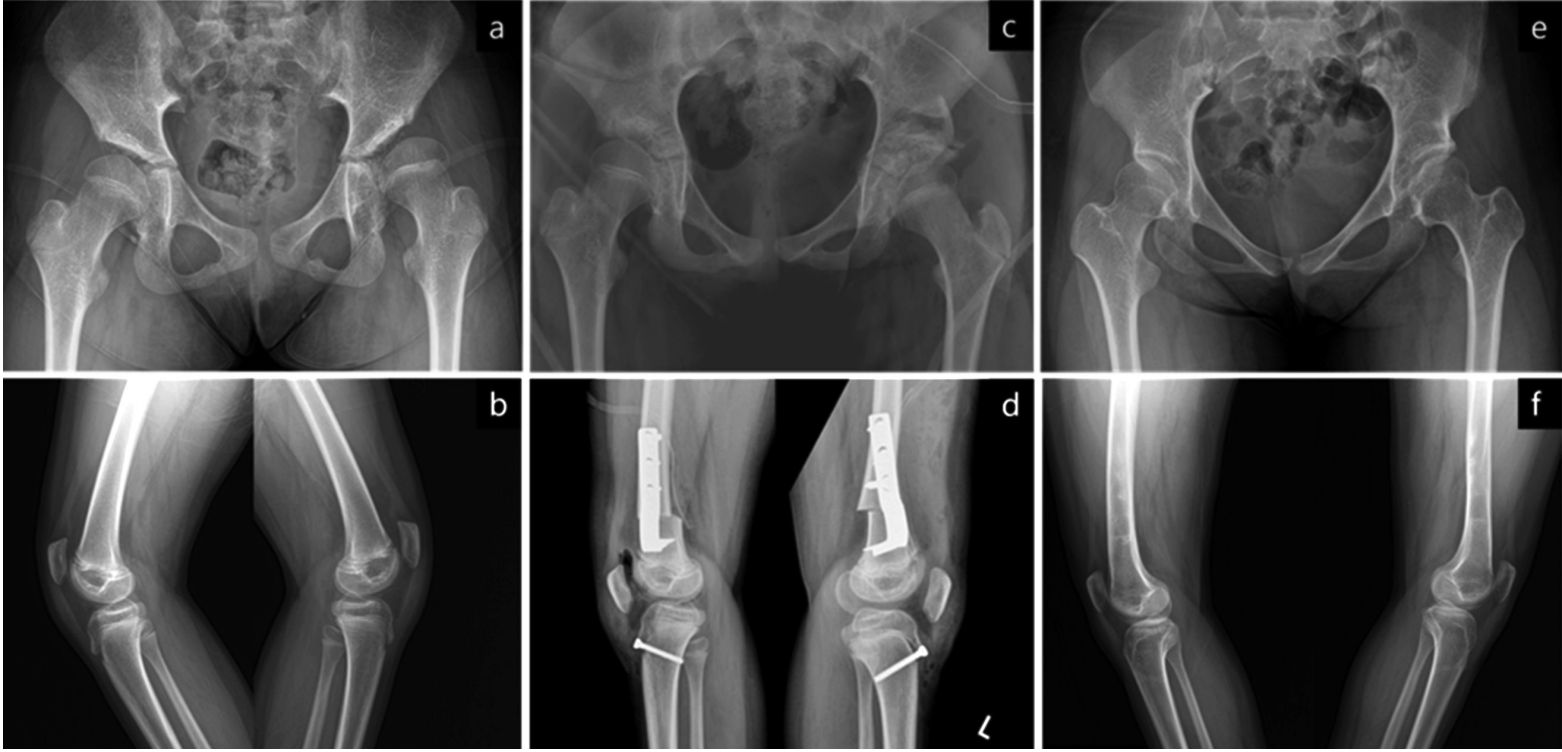
Anteroposterior pelvic radiographs and lateral knee radiographs of a patient in Group C. (a, b) Preoperative radiographs, taken at 11 years and 3 months of age, demonstrate left hip subluxation with a migration percentage of 57%, along with bilateral knee flexion contractures. (c, d) Postoperative radiographs show successful reduction of the femoral head, correction of acetabular dysplasia, and improvement in knee flexion deformities following open reduction, distal femoral derotational and shortening osteotomy, and iliac osteotomy. Additionally, simultaneous procedures included bilateral patellar tendon advancement, tibial tubercle epiphysiodesis, and correction of left planovalgus foot deformity. (e, f) At 17 years of age, the Melbourne Cerebral Palsy Hip Classification Scale was graded as 2. The patient’s preoperative Gross Motor Function Classification System level III remained unchanged.

In Group C, concentric hip reduction was achieved with significant internal rotation and minimal to no abduction (Figure 4). These patients presented with an average knee contracture of 22°, accompanied by marked hamstring tightness. The affected knees were unable to achieve full passive extension [12]. Only minor differences were observed between the unilateral and bilateral popliteal angles (80° and 70°, respectively). The degrees of femoral derotation and the length of shortening were determined based on the required internal rotation for concentric hip reduction, as well as the need to correct fixed knee flexion deformity [13,14]. The average femoral shortening was 1.5 cm (range 1.0 to 2.5). After fixation with a plate and hip extension, the knee achieved full extension or displayed minimal residual contracture. An iliac osteotomy was performed in all hips. Various types of patellar tendon advancement [13] were performed in 12 patients, and additional hamstring lengthenings in 2.

#### Group D: Open reduction and proximal femoral varus derotational osteotomy (FVDO) with or without iliac osteotomy (20 hips in 14 patients)

**Figure 5:**
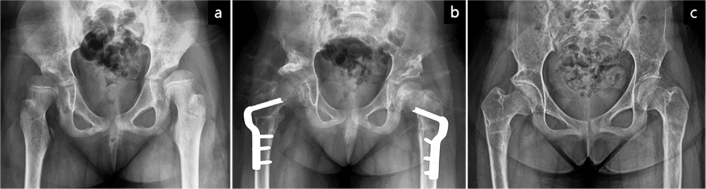
Anteroposterior pelvic radiographs of a patient in Group D. (a) The preoperative radiograph, taken at 9 years and 2 months of age, demonstrates bilateral hip subluxation with migration percentages of 77% on the right side and 54% on the left side. (b) The postoperative radiograph shows successful reduction of the femoral heads and correction of acetabular dysplasia following bilateral open reduction, femoral varus derotational osteotomy, and pelvic osteotomy. Additionally, simultaneous bilateral medial hamstring lengthening and gastrocnemius recession were performed. (c) At 13 years and 10 months of age, the Melbourne Cerebral Palsy Hip Classification Scale was graded as 2 for the right hip and 1 for the left hip. The patient’s preoperative Gross Motor Function Classification System level III remained unchanged.

When the hip was reduced with more than 30° of abduction and internal rotation, an FVDO was performed (Figure 5). If necessary, an extension component was incorporated into the osteotomy. Six patients underwent bilateral reconstructions. In 1 patient, both FVDO and distal femoral extension osteotomy were performed simultaneously to correct the knee flexion deformity. An iliac osteotomy was done on all hips except for 3. Hamstring lengthenings were performed on 9 ipsilateral limbs, and 2 patients underwent lengthenings on both limbs.

**Figure 6:**
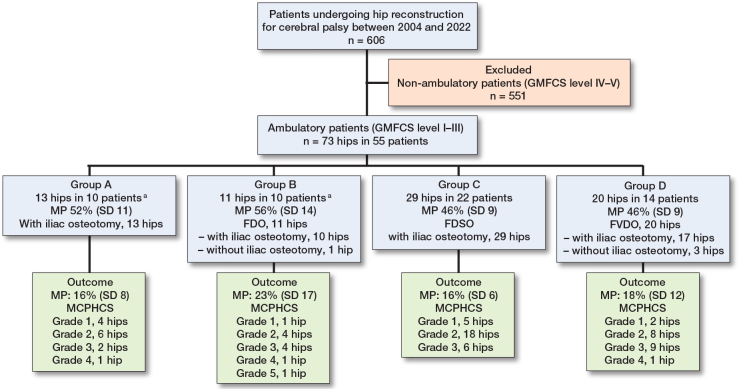
Flow diagram of patient selection with the 4 types of hip reconstruction performed as part of a single-event multilevel surgery. Migration percentage (MP) values for each subgroup are presented for descriptive purposes only. No statistical comparisons were performed between the subgroups. FDO = proximal femoral derotational osteotomy, FDSO = distal femoral derotational and shortening osteotomy, FVDO = proximal femoral varus derotational osteotomy, MCPHCS = Melbourne Cerebral Palsy Hip Classification Scale. ^a^ One patient had separate surgical treatment regimens that were needed for correction of each respective hip.

### Radiographic examinations

To accurately assess altered proximal femur geometry and acetabular deficiency location [15], we utilized preoperative two-dimensional (6 patients) and three-dimensional (47 patients) computed tomography (CT) scans. We measured the neck–shaft angle (cNSA) and femoral anteversion [16]. Additionally, to evaluate acetabular orientation, we assessed the acetabular anteversion angle (AAA) [16] and the axial acetabular index (AAI) [17]. Preoperative, postoperative, and final anteroposterior (AP) pelvic radiographs were analyzed to measure migration percentage, acetabular angle of Sharp (SA), neck–shaft angle (NSA), head-shaft angle, and pelvic obliquity. The presence and severity of avascular necrosis (AVN) [18], as well as limb length inequality, were evaluated using follow-up radiographs. To minimize measurement error, all radiographic parameters were independently measured by 2 fellowship-trained pediatric orthopedic surgeons (KHP and BKP). Intra- and inter-observer reliability was assessed for radiographic and CT measurements (Table 3).

**Table 3:** Intra-observer and inter-observer reliability of radiographic measurements

Variable	Intra-observer reliability ICC (CI)	Inter-observer reliability ICC (CI)
Plain radiograph		
Migration percentage	0.92 (0.87–0.97)	0.94 (0.90–0.97)
Sharp angle	0.86 (0.78–0.94)	0.87 (0.82–0.95)
Neck–shaft angle	0.93 (0.89–0.97)	0.91 (0.86–0.96)
Head–shaft angle	0.89 (0.83–0.96)	0.84 (0.81–0.90)
Pelvic obliquity	0.92 (0.87–0.97)	0.90 (0.85–0.96)
Computed tomography		
Neck–shaft angle	0.80 (0.63–0.87)	0.78 (0.61–0.87)
Femoral anteversion	0.83 (0.69–0.90)	0.79 (0.60–0.88)
Acetabular anteversion angle	0.88 (0.84–0.94)	0.92 (0.88–0.96)
Axial acetabular index	0.85 (0.79–0.91)	0.88 (0.87–0.95)

ICC = intraclass correlation coefficient; CI = 95% confidence interval.

### Outcomes

The Melbourne Cerebral Palsy Hip Classification Scale (MCPHCS [18] Supplementary Figure) was assessed by 3 surgeons, who then reached a consensus on their findings. The overall developmental status of the hip was examined based on the migration percentage, integrity of the Shenton line, femoral head shape, acetabular shape, pelvic obliquity, and hip pain. A satisfactory outcome was defined as MCPHCS grades of 1, 2, or 3, while an unsatisfactory as grade 4, 5, or 6 [19].

### Statistics

Descriptive statistics, Shapiro–Wilk tests, and paired t-tests were performed using SPSS version 25.0 (IBM Corp, Armonk, NY, USA). The linear mixed-effects models and extraction of least-squares means were performed in R version 4.4.1 (R Foundation for Statistical Computing, Vienna, Austria). All hypothesis tests were two-sided, and a P value < 0.05 was considered statistically significant. Normality was evaluated using the Shapiro–Wilk test for all continuous radiographic and CT-derived variables, assessed on raw data separately in subluxated and non-subluxated hips. For the linear mixed-effects models, residual distributions were examined using Quantile–Quantile (Q–Q) plots; no substantial deviations from normality were observed, supporting the adequacy of the model assumptions. Residual Q–Q plots are provided in Supplementary data.

Inter-rater reliability of radiographic measurements was evaluated using intraclass correlation coefficients with 95% confidence intervals (CIs). Longitudinal changes across 3 time points (preoperative, postoperative, and final follow-up) in subluxated hips, including bilateral hips from the same patient, were analyzed using repeated-measures linear mixed-effects models with patient ID as a random intercept. Least-squares means and their CIs in Table 4 were derived from the unadjusted linear mixed-effects model, in which time was the only fixed effect and patient ID was included as a random intercept. Bonferroni correction was applied to the 3 pairwise comparisons (preoperative vs postoperative, postoperative vs final, and preoperative vs final). For non-subluxated hips (1 hip per patient), paired t-tests were used to compare the mean radiographic measurements between the preoperative and final follow-up time points, which are reported as means with standard deviations (SDs). For subluxated hips, mixed-effects models were applied because some patients contributed bilateral hips and had 3 repeated measurements; these results are presented as model-estimated least-squares means with CIs. CT-derived anatomical variables are presented as descriptive statistics, with means and standard deviations (SDs). There were no missing data for the primary radiographic outcomes. Several baseline CT-derived parameters required 3D CT reconstruction and therefore had incomplete data. No imputation was performed.

**Table 4:** Radiographic measurements and pairwise comparisons

Variable	LS mean (CI)	Overall P value	Pairwise comparison	Difference (CI)	P value
Time point					
Migration percentage (%)					
Pre	53.6 (50.5–56.6)	< 0.001	Pre vs Post	38.1 (33.3 to 42.9)	< 0.001
Post	15.5 (12.4–18.5)		Pre vs Final	35.8 (30.9 to 40.6)	< 0.001
Final	17.8 (14.7–20.9)		Post vs Final	–2.3 (–7.2 to 2.5)	0.7
Sharp angle (°)					
Pre	54.3 (53.2–55.4)	< 0.001	Pre vs Post	13.8 (12.2 to 15.5)	< 0.001
Post	40.4 (39.3–41.5)		Pre vs Final	11.7 (10.1 to 13.3)	< 0.001
Final	42.6 (41.5–43.7)		Post vs Final	–2.1 (–3.8 to –0.5)	0.005
Neck–shaft angle (°)					
Pre	148.1 (144.8–151.5)	< 0.001	Pre vs Post	9.4 (6.1 to 12.8)	< 0.001
Post	138.7 (135.4–142.1)		Pre vs Final	10.0 (6.7 to 13.4)	< 0.001
Final	138.1 (134.8–141.4)		Post vs Final	0.6 (–2.7 to 4.0)	1
Head–shaft angle (°)					
Pre	157.9 (155.0–160.9)	< 0.001	Pre vs Post	8.0 (4.1 to 11.9)	< 0.001
Post	150.0 (147.0–152.9)		Pre vs Final	10.8 (6.9 to 14.7)	< 0.001
Final	147.2 (144.2–150.1)		Post vs Final	2.8 (–1.1 to 6.7)	0.3
Pelvic obliquity (°)					
Pre	2.3 (1.7–2.9)	0.003	Pre vs Final	–1.2 (–1.9 to –0.4)	0.003
Final	3.5 (2.9–4.1)		N/A	(.N/A	.N/A

CI = 95% confidence interval.

Least-squares means (LS means) were estimated using an unadjusted linear mixed-effects model with patient ID as a random intercept to account for within-subject correlation. Time (preoperative, postoperative, and final follow-up) was the only fixed effect. Accordingly, LS means represent unadjusted model-based predicted values. Pairwise differences were derived from the same model, and P values were adjusted using the Bonferroni method for 3 comparisons (pre vs post, post vs final, pre vs final).

### Ethics, data sharing, funding, and disclosures

Ethics approval was obtained (IRB No. 4-2018-1227). Data is available from the corresponding author upon reasonable request. This study was partially supported by a faculty research grant from Yonsei University College of Medicine (6-2023-0076). Completed ICMJE disclosure forms are available on the article page, doi: 10.2340/17453674.2026.45513

## Results

The patient selection process is shown in Figure 6. Between 2004 and 2022, 73 consecutive displaced hips in 55 ambulatory patients underwent hip reconstruction by the senior surgeon, and all were included in this study. All hips presented with a femoral head migration percentage (MP) greater than 30%. GMFCS level was II in 27 patients and III in 28. 51 patients had diplegia and 4 hemiplegia. Before surgery, they reported increasing difficulty and fatigue while walking, and 18 patients experienced varying degrees of pain (visual analog scale [VAS] 2–6). 5 patients had undergone previous soft tissue surgeries on the ipsilateral limb, including adductor release, hamstring lengthening, and gastrocnemius–soleus lengthening, either individually or in combination. 18 (33%) patients had bilateral simultaneous hip reconstructions, and 37 unilateral. The mean age at surgery was 9.7 years (SD 2.6). The mean follow-up period was 7.2 years (SD 4.6), and the mean age at the latest follow-up was 17.3 years (SD 4.7).

Qualitative analysis of the acetabular deficiencies revealed anterior deficiency in 20 hips (31%), mid-superior in 29 (45%), posterior in 7 (11%), and global in 8 (13%). The femoral anteversion was 40.3º (SD 11.7) in subluxated hips and 35.4º (SD 13.0) in non-subluxated hips. The cNSA was 141.4º (SD 6.1) in subluxated hips and 138.1º (SD 6.2) in non-subluxated hips. The AAA and AAI were 14.8º (SD 4.9) and 119.8º (SD 12.7) in subluxated hips, and 14.5º (SD 4.5) and 113.4º (SD 12.1) in non-subluxated hips, respectively. These values characterize the baseline anatomical morphology of the subluxated and non-subluxated hips and were not subjected to inferential comparison. All primary radiographic measurements were complete. Missingness occurred only in baseline CT-derived parameters: cNSA and acetabular deficiency measurements were unavailable in 8 patients (9 hips), femoral anteversion in 2 patients (2 hips), and AAA/AAI in 2 patients (3 hips).

The values of the parameters measured on plain films are presented in Table 4. In the subluxated hips, the MP was 53.6% (CI 50.5–56.6) before surgery and 17.8% (CI 14.7–20.9) at the final follow-up (P < 0.001). The SA was 54.3° (CI 53.2–55.3) before surgery and 42.6° (CI 41.5–43.7) at the final follow-up (P < 0.001). Based on the linear mixed-effects model, the estimated mean change from preoperative to final follow-up was –35.8% (CI –40.6 to –30.9) for MP and –11.7° (CI –13.3 to –10.1) for SA. In the non-subluxated hips, the MP was 24.5% (SD 9.2) before surgery and 17.1% (SD 9.9) at the final follow-up (P < 0.001). The SA was 51.0° (SD 15.6) before surgery and 44.7° (SD 5.7) at the final follow-up (P = 0.04).

Before surgery, 51 hips had an MP of 30% to less than 60%, and 21 an MP of 60% to less than 100%. 1 hip was completely dislocated. At the latest follow-up, 69 hips had achieved satisfactory outcomes. The final MCPHCS grades were grade 1 in 12 hips, grade 2 in 36, grade 3 in 21, grade 4 in 3, and grade 5 in 1 hip. 4 hips experienced recurrences of instability. In 2 of these hips, while a significant initial reduction in MP and SA was achieved after surgery, subsequent early allograft resorption led to femoral head migration, resulting in MCPHCS grade 4 classifications. The other 2 hips developed AVN, leading to grade 4 and 5 classifications, respectively. Radiological signs of AVN were observed in a total of 5 hips. By the final follow-up, 3 had remodeled, while 2, with involvement of entire epiphyses, did not completely remodel and were classified as MCPHCS grade 4 and grade 5, respectively. The final MCPHCS grades in the hips that were not initially subluxated were: grade 1 in 13 hips, grade 2 in 12, grade 3 in 10, and grade 4 in 2 hips.

### Outcomes

At the latest follow-up, 47 patients maintained their preoperative GMFCS levels, with 23 classified as level II and 24 as level III. 4 patients improved from level III to II, and 1 improved from level II to I. 3 patients experienced a deterioration from level II to III. Preoperative GMFCS level was maintained or improved in all patients in Groups A and B, 21 of 22 in Group C, and 12 of 14 in Group D; 1 patient in Group C developed subluxation of an initially non-subluxated hip, while 2 patients in Group D declined in walking ability despite having 1 operated hip with MCPHCS grade 2 and 2 hips with grade 3. 6 patients had mild to moderate pain (VAS 2–4). The mean limb length inequality at the final follow-up was 0.9 cm (SD 0.8).

## Discussion

Few studies have specifically evaluated the outcomes of hip reconstruction in ambulatory patients [3,4,9]. However, these studies simply translated techniques originally designed for nonambulatory patients. We aimed to evaluate the outcomes of a tailored hip reconstruction strategy within the SEMLS framework, and we achieved successful MCPHCS outcomes in 69 of 73 hips, indicating that our personalized surgical procedures effectively manage hip displacement within the SEMLS framework.

SEMLS have been utilized to address secondary musculoskeletal problems associated with persistent spasticity and continued growth, with the goal of maintaining or improving gait. Within a SEMLS, procedures for the hip joint are typically limited to psoas and adductor releases and proximal femoral derotational osteotomy to correct in-toeing and scissoring gaits. While a specific surgical treatment strategy for hip displacement within SEMLS has yet to be established, our study demonstrates the effectiveness of a tailored approach.

In spastic hip displacements for the nonambulatory patient, the acetabular deficiency is usually posterosuperior or global [15,17,20]. However, the specific location of the deficiency may vary depending on factors such as the degree of hip displacement and the ambulation level. In our cohort, acetabular deficiency was primarily observed in the mid-superior region, followed by the anterior. A recent study found that the acetabulum is often retroverted in quadriplegic patients with dislocated hips [21], likely due to their predominantly seated posture. In contrast, our observations of acetabular anteversion align with findings from cohorts of diplegic patients [16,22]. The AAI in our displaced hips was lower than that reported in nonambulatory patients [16,22] but higher than that in normally developing hips [17]. While measurable acetabular remodeling can occur following isolated FVDO in select nonambulatory patients [6,23], older ambulatory patients with higher functional demands may benefit more from simultaneous correction of the acetabular dysplasia.

In our cohort, pelvic osteotomy was performed in most hips due to the limited potential for spontaneous remodeling, as the patients were relatively older at the time of surgery (mean age: 9.7 years). Considering the higher functional demands of ambulatory patients, our aim was to minimize the risk of recurrent instability through more definitive correction during the initial operation. Pelvic osteotomy was omitted in only 4 hips, all in patients under 6 years of age with greater expected remodeling capacity. All operated-on hips presented with a preoperative SA greater than 45°, and preoperative CT scans revealed acetabular deficiency in most cases. Although postoperative CT scans were not routinely obtained, the consistent improvements in MP and SA suggest that incorporating pelvic osteotomy contributed to enhanced femoral head coverage in this ambulatory population.

Pronounced alterations in the proximal femoral geometry are commonly seen in CP patients [24]. These may be attributed to variations in the growth of the traction epiphyses at the greater and lesser trochanters, which are influenced by the strength of the hip abductors and spasticity in the adductors and flexors [25]. Because our ambulatory cohort presented with a lesser degree of coxa valga than typically reported in nonambulatory individuals, we were able to forgo varus osteotomy of the femur in more than 70% of cases. Preventing the exacerbation of an abductor lurch is crucial, as proximal femoral varization may lead to further abductor insufficiency and mechanical axis deviation. Additionally, this strategy helps prevent limb length discrepancy in patients with unilateral hip displacement. Although the role of femoral anteversion in hip displacement remains inconclusive in the literature [25], we experienced that increased femoral anteversion was more closely related to hip instability than to an increased NSA, similar to previous observations [10,16,26]. Incorporating a varus component may not always be necessary for ambulatory patients. Relying solely on intraoperative arthrography or the instability maneuver, either before or after FVDO, may not be sufficient for assessing hip displacements in ambulatory patients [27]. The precise indications for open reduction or capsulotomy remain undefined [27,28]. However, we believe that accurate hip positioning, in terms of the degrees of derotation and varization of the femur, should be confirmed through direct visualization of the femoral head after sufficient soft tissue releases and capsulotomy, supplemented by fluoroscopic examination to ensure concentric hip reduction.

40% of our displaced hips (29 hips) were accompanied by severe hamstring tightness and varying degrees of crouch gait. To maintain hip stability and prevent falls, it is essential to address increased knee flexion and hip adduction/knee valgus thrust in the stance phase of gait. Over the last 2 decades, various distal femoral extension osteotomies and patellar tendon advancements have gained popularity [13]. We decided on a technique of distal femoral shortening to simultaneously correct hip displacement and crouch gait. A key prerequisite for this approach was that significant hip abduction for femoral head relocation was not required. This approach allowed for varying degrees of internal rotation of the hip necessary for concentric hip reduction while minimizing the risk of neurovascular compromise. As previously mentioned, increased femoral anteversion was more indicative of hip instability than the increased NSA. Therefore, we believe a distal femoral osteotomy more efficiently corrects the increased knee flexion gaits related to the increased femoral anteversion, without unnecessarily performing a femoral varization and creating biomechanical changes in the coronal plane. We achieved satisfactory hip stability with this method and recommend it for ambulatory patients with both hip displacement and knee flexion deformity in the setting of SEMLS. Femoral shortening, performed to correct crouch gait, may increase anterior pelvic tilt [14], potentially reducing posterior femoral head coverage. In this study, all 29 hips in Group C received iliac osteotomy to address acetabular dysplasia. In this subgroup, the mean preoperative MP and SA were 46.2% and 53.8°, respectively, improving postoperatively to 16.1% and 39.8°. This suggests that iliac osteotomy may help counteract the potential decrease in femoral head coverage that can occur with increased anterior pelvic tilt.

In our series, most patients maintained hip stability at the latest follow-up. However, 4 hips showed poor outcomes, and these recurrences of hip instability were attributed to unexpected postoperative complications, such as early allograft resorption or the development of AVN, rather than to surgical errors. Although Shore et al. stated that MP of > 50% was related to a higher revision rate across all GMFCS levels [8], it has recently been suggested that an MP > 28% at triradiate cartilage closure in ambulatory patients is the primary risk factor for progression to an unsuccessful hip [29]. In our study, all hips classified as grade 1, 2, or 3 had an MP < 28% and a well-developed acetabulum, indicating that these hips are unlikely to experience hip displacement.

### Limitations

First, we did not directly compare our surgical algorithm with conventional hip reconstruction. Future randomized controlled studies are warranted to determine whether hip reconstruction should be staged, with knee, ankle, and foot surgeries performed after achieving hip stability. However, we believe a staged approach might not benefit ambulatory patients with hip displacement. Isolated hip reconstruction in this group could lead to fixed deformities in the lower extremities, potentially impairing ambulation and complicating the subsequent management of these deformities. For patients requiring substantial femoral varization and external rotation, conventional hip reconstruction remained necessary, suggesting staged procedures may still be appropriate for this subgroup. Second, fully instrumented gait analysis results were not included due to incomplete data across all patients. Defining specific 3-dimensional gait patterns is particularly complex in patients with unilateral or bilateral hip displacement, especially when concurrent knee and foot/ankle issues are present. Third, while we observed significant radiographic improvements after surgery, we were unable to correlate these changes with patient-reported functional outcomes. This was due to the lack of a validated tool specifically designed to assess hip-related gait dysfunction in ambulatory patients with CP. Finally, as a retrospective single-arm study, residual confounding cannot be eliminated. Although adjusted analyses were performed, these results should be interpreted only as supportive, and the primary interpretation relies on the unadjusted findings.

### Conclusion

Within the SEMLS framework, our tailored hip reconstruction achieved satisfactory hip outcomes in 69 of 73 hips and resulted in sustained improvement in hip stability at long-term follow-up.

### Supplementary data

The Melbourne Cerebral Palsy Hip Classification Scale, the Gait Outcomes Assessment List, a Supplementary Figure and a Supplementary Table are available as Supplementary data on the article homepage, doi: 10.2340/17453674.2026.45513

## Supplementary Material



## Notes

KBP, HWK: Conceptualization. KHP, BKP, HP, HWK, YHR: Methodology. KHP, BKP, YHR: Analysis, investigation. KHP: Data curation. KHP, HWK: Writing—original draft. IR, KHP, HWK: Writing—review and editing. HWK: Supervision.

Handling co-editors: Ivan Hvid and Philippe Wagner

*Acta* thanks Niels Wisbech Pedersen and other anonymous reviewers for help with peer review of this manuscript.
