# Supplementary material for: Outcomes of hip reconstruction in ambulatory patients with cerebral palsy and spastic hip displacement: a retrospective study of 73 hips in 55 consecutive patients
Source: Acta Orthop. 2026 Feb 23;97:126–35. doi: 10.2340/17453674.2026.45513 (PMC12927440; doi:10.2340/17453674.2026.45513)
Supplement: Supplementary file 1 [file ActaO-97-45513-s1.pdf]

The Melbourne Cerebral Palsy Hip Classification Scale
(Expanded and Revised)

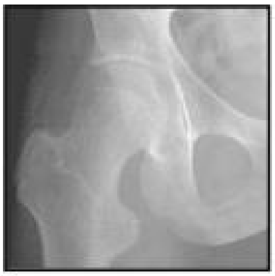
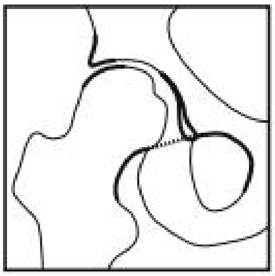

Grade 1: Normal Hip – Migration Percentage <10%

1. Shenton's arch intact
2. Femoral head round (within 2mm using Mose circles)
3. Acetabulum – normal acetabular development with a normal horizontal sourcil, an everted lateral margin and normal tear drop development
4. Pelvic obliquity <5°
5. No degenerative change, no pain

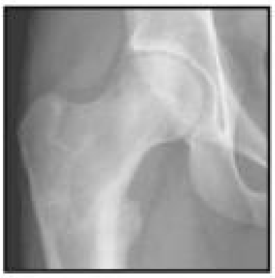
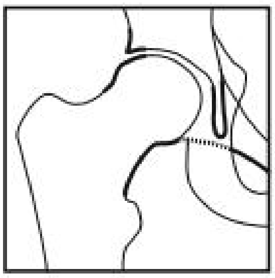

Grade 2: Near Normal Hip – Migration Percentage ≥10% ≤15%

1. Shenton's arch intact
2. Femoral head round or almost round
3. Acetabulum – normal or near normal development
4. Pelvic obliquity <5°
5. Low risk of degenerative change, usually pain free

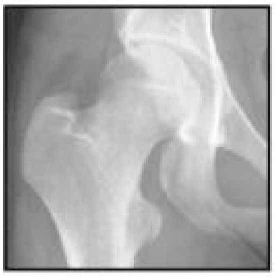
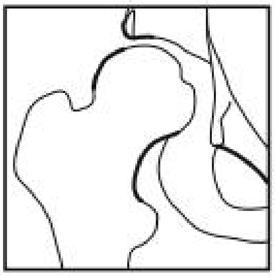

Grade 3: Dysplastic Hip – Migration Percentage >15% ≤30%

1. Shenton's arch intact or broken by ≤5mm
2. Femoral head round or mildly flattened
3. Acetabulum normal or mildly dysplastic including blunting of the acetabular margin and a widened tear drop
4. Pelvic obliquity <10°
5. Low risk of degenerative change, occasionally mild pain

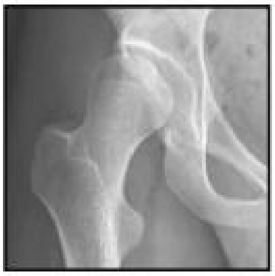
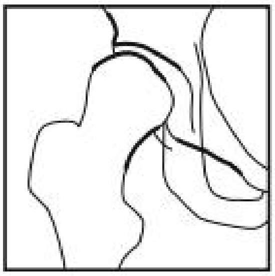

Grade 4: Dysplasia With Mild Subluxation – Migration Percentage >30% <60%

1. Shenton's arch broken by >5mm
2. Femoral head some flattening – Appendix 1
3. Acetabulum dysplastic – Appendix 2
4. Pelvic obliquity variable – Appendix 3
5. Risk of degenerative change, pain variable

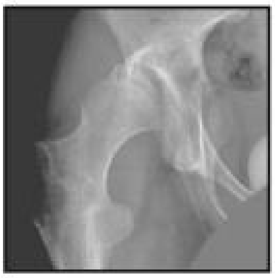
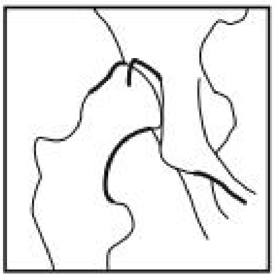

Grade 5: Moderate to Severe Subluxation – Migration Percentage ≥60% <100%

1. Shenton's arch broken by >10mm
2. Femoral head variable deformity – Appendix 1
3. Acetabulum variable deformity – Appendix 2
4. Pelvic obliquity variable – Appendix 3
5. Degenerative change frequent, pain frequent

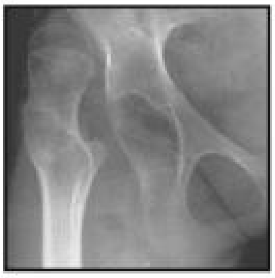
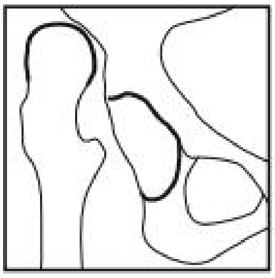

Grade 6: Dislocated Hip – Migration Percentage ≥100%

1. Shenton's arch completely disrupted
2. Femoral head variable deformity – Appendix 1
3. Acetabulum variable deformity – Appendix 2
4. Pelvic obliquity variable – Appendix 3
5. Degenerative change frequent, pain frequent

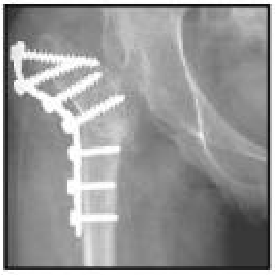
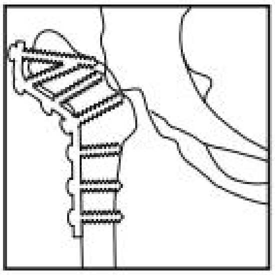

Grade 7: Salvage Surgery

1. Valgus osteotomy
2. Arthrodesis
3. Excision arthroplasty (Castle) ± valgus osteotomy (McHale)
4. Replacement arthroplasty
5. Pain relief following salvage surgery: variable

GOAL

Gait Outcomes Assessment List

Parent Version

Study ID: ______________
REB #: ________________
Event: ________________
Completed by research team

1. We are trying to learn more about how your child walks and what is important to you about their mobility.
2. Please read the instructions on each page carefully.
3. Please answer all questions by circling the number and checking the box that fits best.
4. You may choose to add more items that are important to you at the end of the questionnaire.

For example:

A) Activities of Daily Living & Independence

Consider how your child __usually__ performs each of the following activities.

1) Rate how **easy or difficult** it was for your child to perform each of these activities in the __past 4 weeks__; **AND**
2) Choose how much **assistance** your child required to help them perform these activities; **AND**
3) Select **how important a goal** it is for you to have your child improve in each of the following activities.

During the __past 4 weeks__:	*Extremely Difficult / Impossible*	*Very Difficult*	*Difficult*	*Slightly Difficult*	*Easy*	*Very Easy*	*No problem at all*	LEVEL of ASSISTANCE: TOTAL	MODERATE	MINIMAL OR SUPERVISED	INDEPENDENT	IMPORTANCE of GOAL: Not a goal	Not very important	Fairly important	Very important	Extremely important
**1. Getting in and out of bed**	0	1	2	3	4	(5)	6	0	(1)	2	3	○	○	○	☒	○

In the above example, **getting in and out of bed** was rated as ***very easy;*** *required* a ***moderate level of assistance;*** & improving this is a ***very important*** goal.

B) Gait Function & Mobility

Consider how your child __usually__ performs each of the following activities.

1) Rate how **easy or difficult** it was for your child to perform each of these activities in the __past 4 weeks__; **AND**
2) Choose what **walking aid** your child required to help them perform these activities; **AND**
3) Select **how important a goal** it is for you to have your child improve in each of the following activities.

During the __past 4 weeks__:	*Extremely Difficult / Impossible*	*Very Difficult*	*Difficult*	*Slightly Difficult*	*Easy*	*Very Easy*	*No problem at all*	WALKING AID REQUIRED: WHEELCHAIR	WALKER	TWO CANES OR CRUTCHES	ONE CANE /CRUTCH HAND SUPPORT	INDEPENDENT	IMPORTANCE of GOAL: Not a goal	Not very important	Fairly important	Very important	Extremely important
**11. Walking for more than 250 meters (about 2 blocks or 2 football fields)**	(0)	1	2	3	4	5	6	0	(1)	2	3	4	○	○	☒	○	○

In the above example, **walking for 250 meters** was rated as ***extremely difficult***; done with the aid of a ***walker***; & improving this activity is a ***fairly important*** goal.

Relationship to Patient: __________________________   Date of Completion (dd/mm/yyyy): ____ / ____ / _________

A) Activities of Daily Living & Independence								LEVEL of ASSISTANCE				IMPORTANCE of GOAL				
Consider how your child **usually** performs each of the following activities.<br>1) Rate how **easy or difficult** it was for your child to perform each of these activities in the **past 4 weeks**; **AND**<br>2) Choose how much **assistance** your child required to help them perform these activities; **AND**<br>3) Select **how important a goal** it is for you to have your child improve in each of the following activities.																
**During the past 4 weeks:**	***Extremely Difficult / Impossible***	***Very Difficult***	***Difficult***	***Slightly Difficult***	***Easy***	***Very Easy***	***No problem at all***	**TOTAL**	**MODERATE**	**MINIMAL OR SUPERVISED**	**INDEPENDENT**	Not a goal	Not very important	Fairly important	Very important	Extremely important
**1. Getting in and out of bed**	0	1	2	3	4	5	6	0	1	2	3	○	○	○	○	○
**2. Getting in and out of a chair (or wheelchair)**	0	1	2	3	4	5	6	0	1	2	3	○	○	○	○	○
**3. Standing at a sink or counter**	0	1	2	3	4	5	6	0	1	2	3	○	○	○	○	○
**4. Washing/bathing his/her self (eg. shower or tub)**	0	1	2	3	4	5	6	0	1	2	3	○	○	○	○	○
**5. Getting dressed**	0	1	2	3	4	5	6	0	1	2	3	○	○	○	○	○
**6. Carrying an object while walking (eg. toy, book, cell or mobile phone)**	0	1	2	3	4	5	6	0	1	2	3	○	○	○	○	○
**7. Opening a door**	0	1	2	3	4	5	6	0	1	2	3	○	○	○	○	○
**8. Picking up an object off the floor**	0	1	2	3	4	5	6	0	1	2	3	○	○	○	○	○
**9. Getting in and out of a vehicle (eg. car, van or bus)**	0	1	2	3	4	5	6	0	1	2	3	○	○	○	○	○

B) Gait Function & Mobility

Consider how your child **usually** performs each of the following activities.

1) Rate how **easy or difficult** it was for your child to perform each of these activities in the **past 4 weeks**; **AND**
2) Choose what **walking aid** your child required to help them perform these activities; **AND**
3) Select **how important a goal** it is for you to have your child improve in each of the following activities.

During the past 4 weeks:	*Extremely Difficult / Impossible*	*Very Difficult*	*Difficult*	*Slightly Difficult*	*Easy*	*Very Easy*	*No problem at all*	WALKING AID REQUIRED: WHEELCHAIR	WALKER	TWO CANES OR CRUTCHES	ONE CANE /CRUTCH/HAND SUPPORT/ RAILING OR WALL	INDEPENDENT	IMPORTANCE of GOAL: Not a goal	Not very important	Fairly important	Very important	Extremely important
**10. Walking for more than 250 meters (around 2 blocks or 2 football fields)**	0	1	2	3	4	5	6	0	1	2	3	4	○	○	○	○	○
**11. Getting around at school (indoors)**	0	1	2	3	4	5	6	0	1	2	3	4	○	○	○	○	○
**12. Getting around at home**	0	1	2	3	4	5	6	0	1	2	3	4	○	○	○	○	○
**13. Walking for more than 15 minutes**	0	1	2	3	4	5	6	0	1	2	3	4	○	○	○	○	○
**14. Walking faster than usual to keep up with others**	0	1	2	3	4	5	6	0	1	2	3	4	○	○	○	○	○
**15. Stepping around or avoiding obstacles**	0	1	2	3	4	5	6	0	1	2	3	4	○	○	○	○	○
**16. Going up and down stairs**	0	1	2	3	4	5	6	0	1	2	3	4	○	○	○	○	○
**17. Going up and down slopes**	0	1	2	3	4	5	6	0	1	2	3	4	○	○	○	○	○
**18. Walking on uneven ground (rough, rocky, sandy)**	0	1	2	3	4	5	6	0	1	2	3	4	○	○	○	○	○
**19. Walking on slippery surfaces (wet or icy)**	0	1	2	3	4	5	6	0	1	2	3	4	○	○	○	○	○

C) Pain / Discomfort / Fatigue

Consider each of the following items.

1) Rate how **often** your child experienced pain or discomfort or tiredness in the **past 4 weeks**; **AND**
2) Choose how **severe** the pain or discomfort was; **AND**
3) Select **how important a goal** it is for you to reduce your child's pain or discomfort or tiredness in each of the following.

**During the past 4 weeks:**	*Every Day*	*Very Often (nearly every day)*	*Fairly Often (2 to 3 times a week)*	*A Few Times (once a week)*	*Once or Twice*	*None of the Time*	**INTENSITY** SEVERE	MODERATE	MILD	NONE	**IMPORTANCE of GOAL** Not a goal	Not very important	Fairly important	Very important	Extremely important
**20. Pain or discomfort in the feet or ankles**	0	1	2	3	4	5	0	1	2	3	○	○	○	○	○
**21. Pain or discomfort in the knees**	0	1	2	3	4	5	0	1	2	3	○	○	○	○	○
**22. Pain or discomfort in the thighs or hips**	0	1	2	3	4	5	0	1	2	3	○	○	○	○	○
**23. Pain or discomfort in the back**	0	1	2	3	4	5	0	1	2	3	○	○	○	○	○
**24. Feeling tired while walking**	0	1	2	3	4	5	0	1	2	3	○	○	○	○	○
**25. Feeling tired during any other physical activities that he/she usually enjoys (eg. swimming, running, horseback riding or other sport)**	0	1	2	3	4	5	0	1	2	3	○	○	○	○	○
**Other pain:** ________________	0	1	2	3	4	5	0	1	2	3	○	○	○	○	○

D) Physical Activities, Games & Recreation

IMPORTANCE of GOAL

Consider how your child **usually** performs each of the following activities.

1) Rate how **easy or difficult** it was for your child to typically perform each of these activities in the **past year**; **AND**
2) Select **how important a goal** it is for you to have your child improve in each of the following activities.

During the past year:	*Extremely Difficult / Impossible*	*Very Difficult*	*Difficult*	*Slightly Difficult*	*Easy*	*Very Easy*	*No problem at all*	My child did not have the chance to do this activity in the past year	Not a goal	Not very important	Fairly important	Very important	Extremely important
**26. Running**	0	1	2	3	4	5	6	☐	○	○	○	○	○
**27. Participating in gliding sports (eg. skating, rollerblading, skiing, skate/snowboarding)**	0	1	2	3	4	5	6	☐	○	○	○	○	○
**28. Riding a bike or tricycle (with or without training wheels)**	0	1	2	3	4	5	6	☐	○	○	○	○	○
**29. Swimming**	0	1	2	3	4	5	6	☐	○	○	○	○	○
**30. Participating in sports that require running (eg. soccer, baseball, football, track)**	0	1	2	3	4	5	6	☐	○	○	○	○	○
**31. Participating in sports that require jumping (eg. basketball, volleyball)**	0	1	2	3	4	5	6	☐	○	○	○	○	○
**32. Participating in dance or martial arts (eg. karate, judo, taekwondo)**	0	1	2	3	4	5	6	☐	○	○	○	○	○
**33. Climbing (eg. ladder or playground equipment)**	0	1	2	3	4	5	6	☐	○	○	○	○	○
**Other recreational or sporting activity: ________________**	0	1	2	3	4	5	6	☐	○	○	○	○	○

E) Gait Appearance

Consider how your child **usually** walks.

1) Rate how **much of a problem** your child experienced with each of the following in the **past 4 weeks**; **AND**
2) Select **how important a goal** it is for you to have your child improve in each of the following.

During the past 4 weeks:	*Extremely Difficult / Impossible*	*Very Difficult*	*Difficult*	*Slightly Difficult*	*Easy*	*Very Easy*	*No problem at all*	Not a goal	Not very important	Fairly important	Very important	Extremely important
**34. Walking with his/her feet flat on the ground**	0	1	2	3	4	5	6	○	○	○	○	○
**35. Walking taller or more upright (less crouched or bent at the knees)**	0	1	2	3	4	5	6	○	○	○	○	○
**36. Walking with his/her feet pointing straight ahead**	0	1	2	3	4	5	6	○	○	○	○	○
**37. Walking without dragging his/her feet**	0	1	2	3	4	5	6	○	○	○	○	○
**38. Walking without tripping and falling**	0	1	2	3	4	5	6	○	○	○	○	○
**39. Wearing footwear of his/her choice (eg. shoes, boots, sandals)**	0	1	2	3	4	5	6	○	○	○	○	○
**Other aspect of your child's walking: _______________**	0	1	2	3	4	5	6	○	○	○	○	○

IMPORTANCE of GOAL: Not a goal / Not very important / Fairly important / Very important / Extremely important

F) Use of Braces and Assistive Devices

Consider each of the following items.

1) Rate how **you felt** about your child using each of the following in the **past 4 weeks**; **AND**
2) Select **how important a goal** it is for you to have your child to reduce or eliminate their use of these devices.

**During the past 4 weeks:**	***Very Unhappy***	***Unhappy***	***Neither Happy nor Unhappy***	***Happy***	***Very Happy***	IMPORTANCE of GOAL: Not a goal	Not very important	Fairly important	Very important	Extremely important		
**40. Wearing braces or orthotics (eg. AFO)**	0	1	2	3	4	○	○	○	○	○	☐	My child has not been prescribed to use braces, lifts or orthotics.
											☐	My child chooses not to use his/her prescribed braces, lifts or orthotics.
**41. Using a walking aide (eg. walker, stick, cane, crutches)**	0	1	2	3	4	○	○	○	○	○	☐	My child does not use any walking aides.
**42. Using a wheelchair**	0	1	2	3	4	○	○	○	○	○	☐	My child does not use a wheelchair.

G) Body Image & Self-Esteem

Consider each of the following items.

1) Rate how **your child feels** about each of the following in the **past 4 weeks**; **AND**
2) Select **how important a goal** it is for you to have your child improve in each of the following.

**During the past 4 weeks:**	***Very Unhappy***	***Unhappy***	***Neither Happy nor Unhappy***	***Happy***	***Very Happy***	IMPORTANCE of GOAL: Not a goal	Not very important	Fairly important	Very important	Extremely important
**43. The shape and position of his/her legs**	0	1	2	3	4	○	○	○	○	○
**44. The shape and position of his/her feet**	0	1	2	3	4	○	○	○	○	○
**45. The symmetry of his/her legs (in length and size)**	0	1	2	3	4	○	○	○	○	○
**46. The way he/she gets around compared with others**	0	1	2	3	4	○	○	○	○	○
**47. The way others feel about how he/she gets around**	0	1	2	3	4	○	○	○	○	○
**48. How he/she is treated by others**	0	1	2	3	4	○	○	○	○	○

Other Goals

If there are any other goals (long or short term) that we have missed, please list them below **AND** Select **how important a goal** it is for you to have your child improve in each.	IMPORTANCE of GOAL				
Other Goals:	Not a goal	Not very important	Fairly important	Very important	Extremely important
**1.**	◯	◯	◯	◯	◯
**2.**	◯	◯	◯	◯	◯
**3.**	◯	◯	◯	◯	◯
**4.**	◯	◯	◯	◯	◯
**5.**	◯	◯	◯	◯	◯

Comments & Suggestions

Thank You

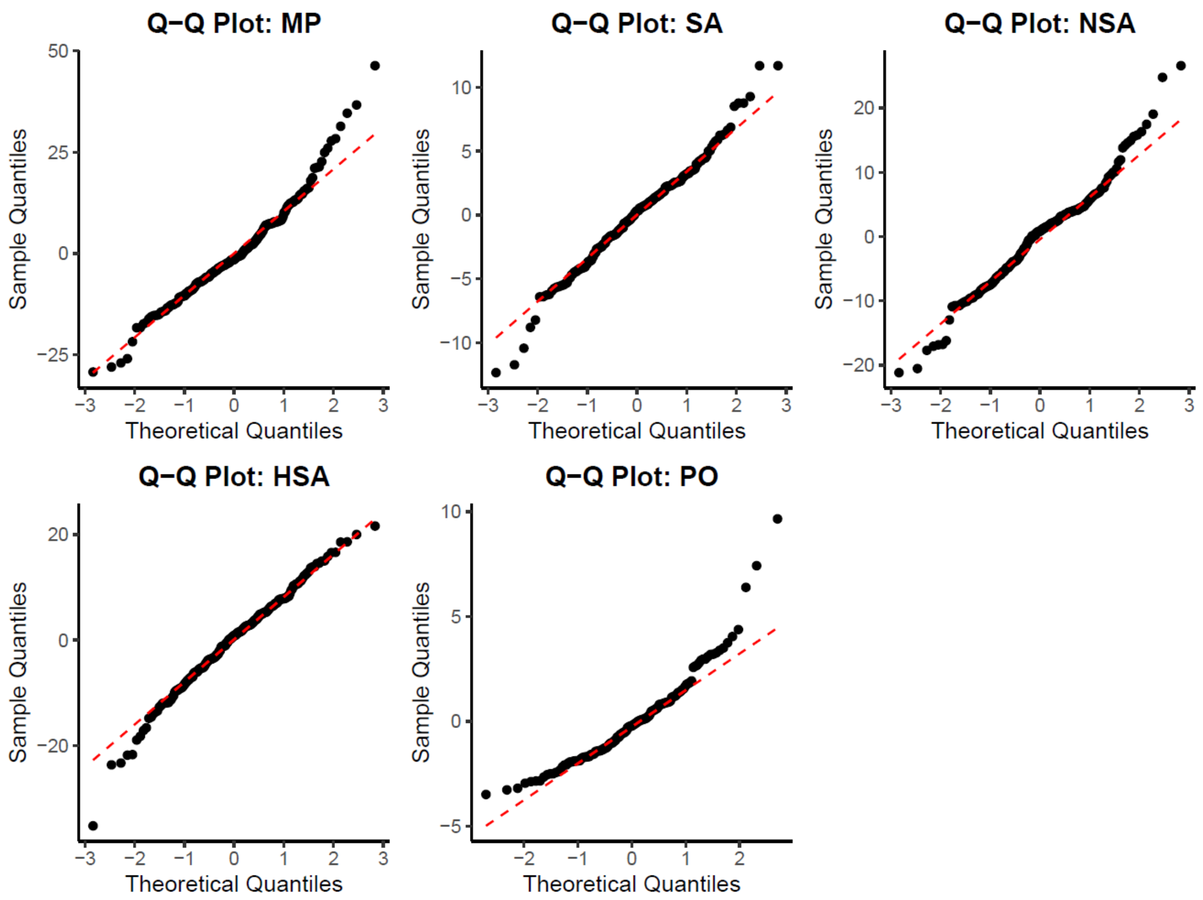

Supplementary Figure. Normality assessment of residuals from the linear mixed-effects model.

MP = migration percentage; SA = Sharp angle; NSA = neck shaft angle; HSA = head shaft angle; PO = pelvic obliquity.

Q–Q (Quantile–Quantile) plots for the model residuals of each radiographic outcome. No substantial deviations from normality were observed.

Supplementary Table. Adjusted radiographic measurements and adjusted pairwise comparisons

Variable	Time point	LS-mean (CI)	Overall P value	Pairwise comparison	Difference (CI)	P value
Migration percentage (%)			<0.001			
	Pre	54.4 (51.2–57.5)		Pre *vs* Post	38.1 (33.3 to 42.9)	<0.001
	Post	16.3 (13.1–19.5)		Pre *vs* Final	35.8 (30.9 to 40.6)	<0.001
	Final	18.6 (15.4–21.8)		Post *vs* Final	–2.33 (–7.14 to 2.48)	0.7
Sharp angle (°)			<0.001			
	Pre	54.7 (53.5–55.8)		Pre *vs* Post	13.8 (12.2 to 15.5)	<0.001
	Post	40.8 (39.7–41.9)		Pre *vs* Final	11.7 (10.1 to 13.3)	<0.001
	Final	43.0 (41.8–44.1)		Post *vs* Final	–2.13 (–3.75 to –0.52)	0.005
Neck shaft angle (°)			<0.001			
	Pre	147.9 (145.2–150.7)		Pre *vs* Post	9.40 (6.04 to 12.8)	<0.001
	Post	138.5 (135.8–141.3)		Pre *vs* Final	10.0 (6.67 to 13.4)	<0.001
	Final	137. 9 (135.1–140.7)		Post *vs* Final	0.64 (–2.73 to 4.00)	>0.9
Head shaft angle (°)			<0.001			
	Pre	158.1 (155.4–160.9)		Pre *vs* Post	7.96 (4.08 to 11.9)	<0.001
	Post	150.2 (147.4–152.9)		Pre *vs* Final	10.8 (6.88 to 14.7)	<0.001
	Final	147.4 (144.6 –150.1)		Post *vs* Final	2.80 (–1.09 to 6.68)	0.3
Pelvic obliquity (°)			0.003			
	Pre	2.28 (1.62 to 2.94)		Pre *vs* Final	–1.16 (–1.90 to –0.42)	0.003
	Final	3.44 (2.78 to 4.10)		N/A	N/A	N/A

CI = 95% confidence interval.

Adjusted least-squares means (LS-means) and pairwise differences were estimated using a linear mixed-effects model with patient ID as a random intercept. Age, sex, and preoperative GMFCS level were included as covariates. Time (preoperative, postoperative, and final follow-up) was modeled as the fixed effect. P values for pairwise comparisons were adjusted using the Bonferroni method (Pre vs Post, post vs Final, Pre vs Final).
